# Supplementary material for: The CarSR two-component system directly controls radD expression as a global regulator that senses bacterial coaggregation in Fusobacterium nucleatum
Source: J Bacteriol. 2025 May 21;207(6):e00529-24. doi: 10.1128/jb.00529-24 (PMC12186487; doi:10.1128/jb.00529-24)
Supplement: Supplemental material — Fig. S1 to S8 and Table S1 to S3. [file jb.00529-24-s0001.docx]

**The CarRS Two-Component System Directly Controls Surface Adhesin RadD Expression as a Global Regulator that Senses Bacterial Coaggregation in Fusobacterium nucleatum**

**Bibek G C^†^ & Chenggang Wu^†^**

*Department of Microbiology & Molecular Genetics, the University of Texas Health Science Center, Houston, TX, USA*

**^†^** To whom correspondence should be addressed. Tel. (+1) 713 500 5437; E-mail: [bibek.gc@uth.tmc.edu](mailto:bibek.gc@uth.tmc.edu) and [chenggang.wu@uth.tmc.edu](mailto:chenggang.wu@uth.tmc.edu)

Running Title: CarRS Senses Coaggregation and Controls RadD Expression

Keywords: *Fusobacterium nucleatum*, RadD, coaggregation, two-component system, CarRS, ChIP-seq

**
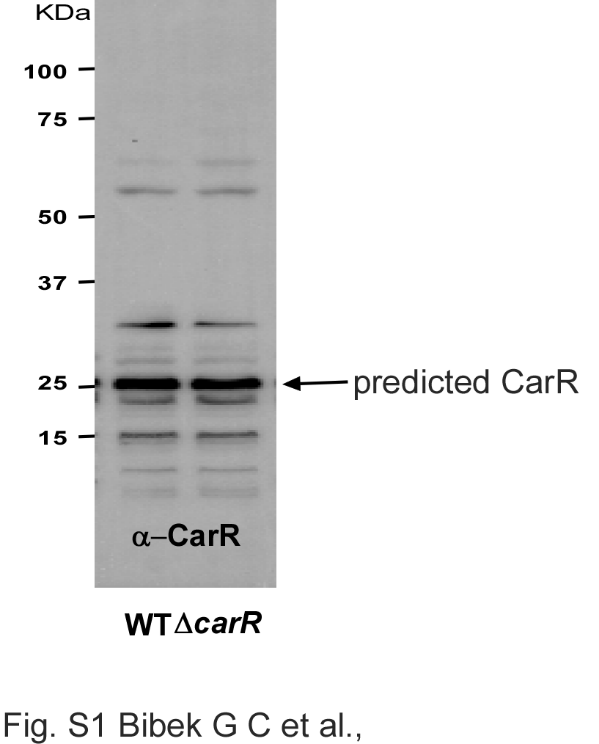
**

**Fig. S1: Lack of specificity of the generated polyclonal antibody for CarR.** The antibody fails to detect a specific band distinguishing wild type (WT) from carR mutant cells, with several nonspecific bands observed. The predicted molecular weight of CarR in WT strain is 25.7KDa.

**Experimental procedure:** Wild-type (WT) and ∆*carR* strains were grown in TSC medium under anaerobic conditions until reaching an OD600 of ~1.0. Cells from 1 mL of each culture were harvested, and the pellets were washed twice with water before being resuspended in sodium dodecyl sulfate (SDS) sample buffer. The samples were boiled for 10 minutes and proteins were resolved on a 4%–20% Tris-glycine gradient SDS-PAGE (BIO-RAD, #456109). Proteins were then transferred onto a PVDF membrane and probed with rabbit anti-CarR antibody (1:500 dilution). Polyclonal goat anti-rabbit IgG conjugated with IRDye 680LT (#92668021; LI-COR Biosciences, USA) was used as the secondary antibody at a 1:5000 dilution.

**Fig.S2**

**
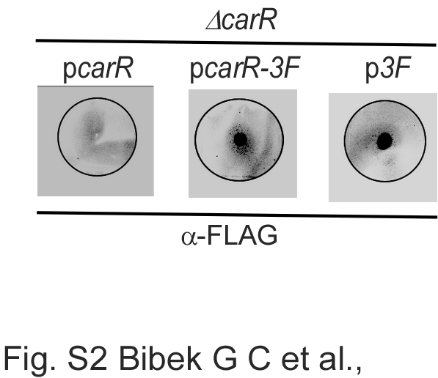
**

**Fig S2: Dot blot analysis of FLAG and 3×FLAG-tagged CarR expression.**

**Dot Blot Assay Procedure:** Cells from *ΔcarR* strains carrying plasmids p*carR*, p*carR*-3F, and p3F were grown to an OD600 of ~1.0, harvested, resuspended in SDS sample buffer, and boiled for 10 minutes. A volume of 50 µL from each sample was spotted onto a nitrocellulose membrane, air-dried, and blocked with 3% non-fat dry milk in PBS-T for 1 hour. The membrane was then incubated overnight at 4°C with rabbit anti-FLAG M2 antibody (#2368, Cell Signaling Technology) at a 1:3000 dilution, followed by three PBS-T washes. A secondary antibody conjugated to an IR dye was applied for 1 hour at room temperature, and the membrane was washed again before detecting the signal using fluorescence imaging. This assay validated the expression of FLAG-tagged CarR, which was not resolvable by SDS-PAGE, through specific FLAG detection (Fig. S2).

**Fig. S3**

**
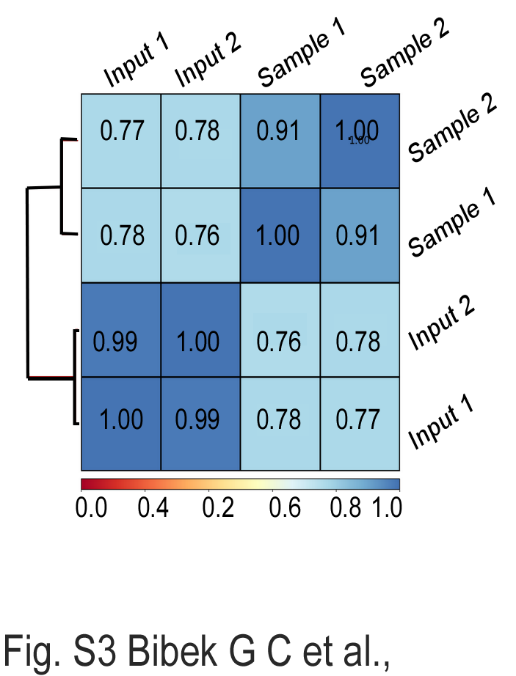
**

**Fig. S3: Correlation heatmap demonstrating ChIP-seq reproducibility**. High correlation coefficients (close to 1.0) between two IP samples and two input controls indicate strong consistency across biological replicates. Heatmap showing the Pearson correlation of ChIP-seq signal intensities between biological replicates (Sample 1 and Sample 2) and corresponding inputs (Input 1 and Input 2). Wild-type cells were subjected to chromatin immunoprecipitation using specific antibodies, and sequencing libraries were prepared from the immunoprecipitated DNA (ChIP) and input DNA (control). Sequencing reads were mapped to the reference genome, and correlation analysis was performed to evaluate reproducibility between samples and inputs. High correlation values indicate consistent and reproducible ChIP-seq results across replicates.

**Fig. S4**

**
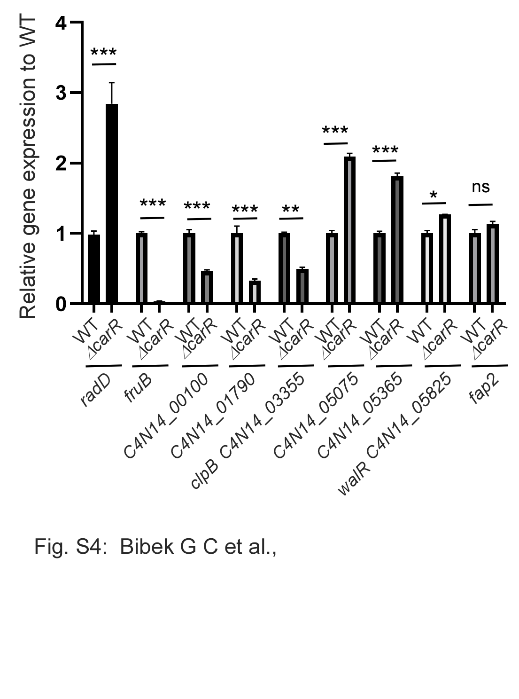
**

**Fig. S4: Validation of ChIP-seq results through quantitative RT-PCR.** The expression of selected genes detected by ChIP-seq but not RNA-seq, including *C4N14_00100* (autotransporter), *C4N14_01790* (glutaredoxin), *clpB*, *C4N14_05075* (*ompA*), *C4N14_05365* (ABC transporter substrate-binding protein), and *C4N14_08250* (*walR*, a response regulator of a WalRK-like TCS), was analyzed in wild-type (WT) and ∆*carR* strains. Quantitative RT-PCR confirmed significant differential expression of these genes, consistent with *carR*-mediated regulation. *Fap2*, another major adhesion protein, showed no differential expression, as it was not detected by either RNA-seq or ChIP-seq, indicating it is not regulated by *carR*. Data are shown as relative gene expression normalized to the WT strain, with *gyrA* as the internal control. Statistical significance: ***p < 0.001; **p < 0.01; *p < 0.05; ns: not significant.

**Experimental procedure:** Wild-type (WT) and ∆*carR* strains were grown to an OD600 of ~0.7, and cells were harvested by centrifugation at 10,000 × g for 5 minutes at 4°C. Cell pellets were resuspended in 1 mL TRIzol reagent (Ambion) and lysed using 0.1-mm silicon beads (MP Bio) in a bead-beater at maximum speed for 1 minute. Total RNA was extracted using the Direct-zol RNA MiniPrep kit (Zymo Research), followed by DNase I treatment to remove genomic DNA. cDNA was synthesized from RNA using SuperScript III reverse transcriptase (Invitrogen) with random primers. Quantitative PCR (qPCR) was performed using iTaq SYBR Green Supermix (Bio-Rad) with primers targeting *radD*, *fruA*, *C4N14_00100* (autotransporter), *C4N14_01790* (glutaredoxin), *clpB*, *C4N14_05075* (*ompA*), *C4N14_05365* (ABC transporter substrate-binding protein), *C4N14_08250* (*walR*), and *fap2* (primer sequences in Table S2). Reactions were carried out in a Bio-Rad CFX96 real-time PCR detection system, and relative gene expression was calculated using the 2^–ΔΔCt method normalized to the *gyrB* housekeeping gene. All experiments were validated in biological duplicates with three technical replicates each. This procedure confirmed the differential expression of genes detected by ChIP-seq but not RNA-seq and validated *carR*-mediated regulation.

**Fig. S5**

**
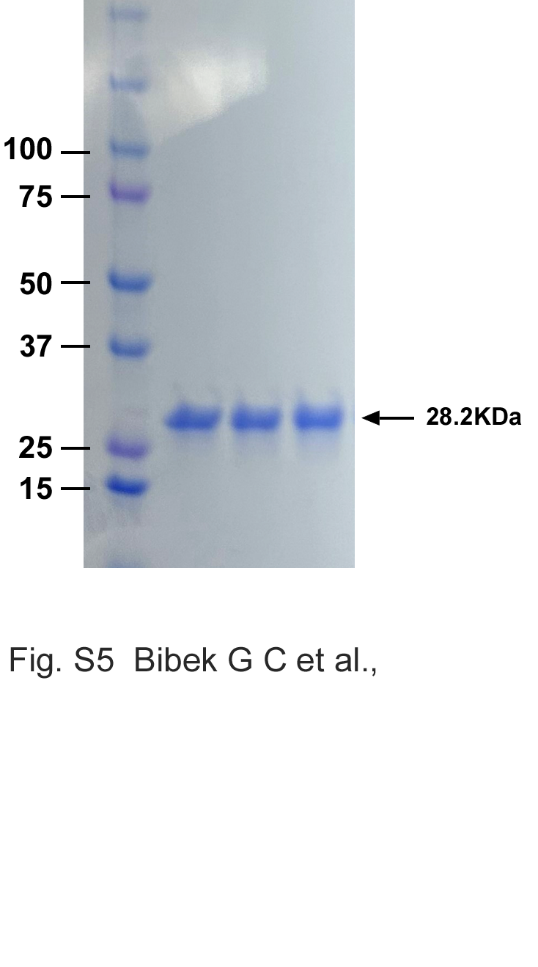
**

**Fig. S5: Purification of full-length CarR protein.** SDS-PAGE analysis of purified full-length CarR expressed in *E. coli*, with the gel stained using Coomassie Brilliant Blue. The arrow indicates the expected molecular weight of CarR (28.2 kDa). The high-quality purified CarR protein was used in electrophoretic mobility shift assays (EMSAs) to validate its direct binding to the *radABCD* promoter.

**Experimental Procedure:** Full-length *carR* was cloned into a pET expression vector and transformed into *E. coli* BL21(DE3) cells for protein expression. Cells were grown in LB medium at 37°C to an OD600 of ~0.6, induced with 0.5 mM IPTG, and incubated at 18°C overnight. Harvested cells were resuspended in lysis buffer (50 mM Tris-HCl pH 8.0, 300 mM NaCl, 10 mM imidazole) and lysed by sonication. After clarification by centrifugation at 12,000 × g for 20 minutes at 4°C, the supernatant was loaded onto a nickel-nitrilotriacetic acid (Ni-NTA) affinity column (Qiagen) for His-tag purification. The column was washed with buffer containing 20 mM imidazole, and CarR was eluted using a buffer containing 250 mM imidazole. Eluted protein was further purified by size-exclusion chromatography on a Superdex 200 column equilibrated with 50 mM Tris-HCl (pH 8.0) and 150 mM NaCl. SDS-PAGE evaluated protein purity, and the gel was stained with Coomassie Brilliant Blue to confirm the expected molecular weight and purity of CarR. The purified protein was concentrated and stored at –80°C for EMSA analysis.

**Fig. S6**

**
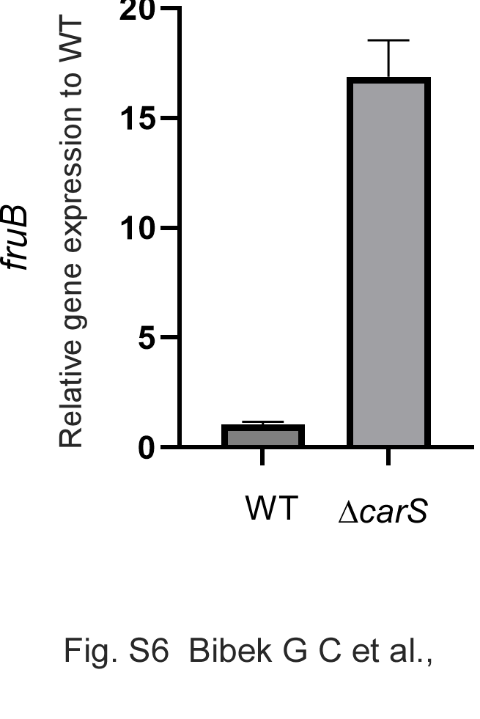
**

**Fig. S6: Upregulation of *fruB* expression in the ∆*carS* mutant.** Quantitative RT-PCR analysis of *fruB* gene expression in wild-type (WT) and ∆*carS* strains. Relative gene expression was calculated using the 2^–ΔΔCt method and normalized to the *gyrB* housekeeping gene. The results show significantly increased *fruB* expression in the ∆*carS* mutant compared to WT, indicating that CarS represses *fruRBA* transcription. These findings are consistent with luciferase activity data, further supporting CarS-mediated repression of *fruRBA* expression. Data are presented as mean ± standard deviation (SD) from biological duplicates, each performed in triplicate.

**Fig. S7**

**
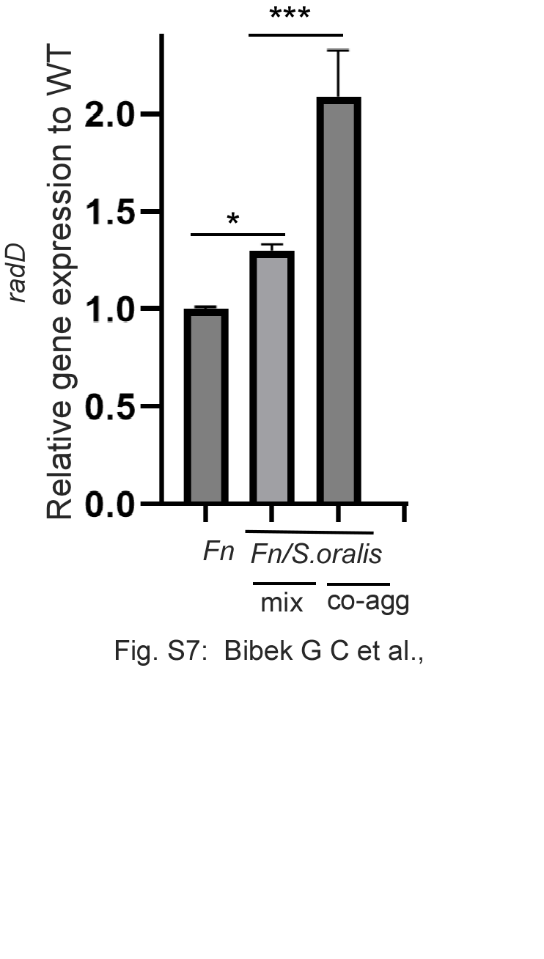
**

**Fig. S7: Coaggregation-dependent upregulation of *radD* expression in *F. nucleatum* during interaction with *S. oralis*.** Quantitative RT-PCR analysis of *radD* gene expression in *F. nucleatum* (Fn) alone, mixed with *S. oralis* without physical interaction (mix), and during coaggregation with *S. oralis* (co-agg). Relative gene expression was calculated using the 2^–ΔΔCt method and normalized to the *gyrB* housekeeping gene. *radD* expression significantly increased during coaggregation with *S. oralis*, indicating that coaggregation-dependent *radD* expression may be a universal feature of *F. nucleatum* in interactions with Gram-positive partners. Data represents the mean ± standard deviation (SD) from biological duplicates, each performed in triplicate. Statistical significance: ***p < 0.001; *p < 0.05.

**
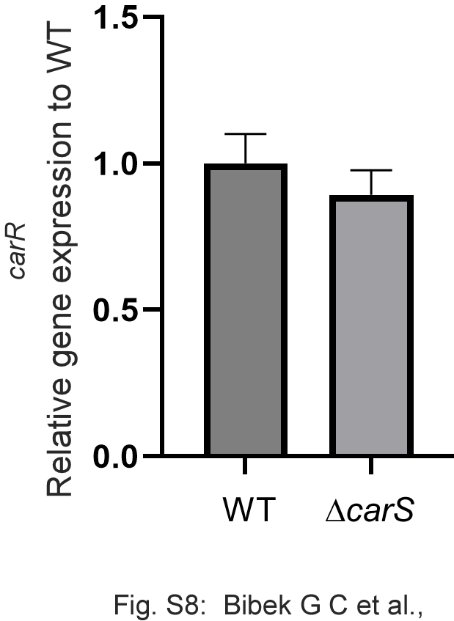
**

**Fig. S8: Deletion of CarS does not affect *carR* expression.** Quantitative RT-PCR analysis of *carR* gene expression in wild-type (WT) and ∆*carS* strains. Relative gene expression was calculated using the 2^–ΔΔCt method and normalized to the *gyrB* housekeeping gene. The results show no change of *carR* expression in the ∆*carS* mutant compared to WTData are presented as mean ± standard deviation (SD) from biological duplicates, each performed in triplicate.

**Supporting Tables**

**Table S1:** Bacterial strains and plasmids used in this study.

| **Strain & Plasmid** | **Description** | | | **Reference** |
| --- | --- | --- | --- | --- |
| **Strains** |  | | |  |
| *Fn* subsp. *nucleatum* 23726 | Parental strain (wild-type strain) | | | From ATCC |
| *Fn* subsp. *nucleatum* CW1 | ∆*galK*; an isogenic derivative of 23726 | | | (1) |
| *Fn* subsp. *nucleatum* CW4 | ∆*radD*; an isogenic derivative of CW1 | | | (2) |
| *Fn* subsp. *nucleatum* CW7 | ∆*carR*; an isogenic derivative of CW1 | | | (2) |
| *Fn* subsp. *nucleatum* CW7c1  *Fn* subsp. *nucleatum* CW7c2  *Fn* subsp. *nucleatum* CW7c3  *Fn* subsp. *nucleatum* CW7c4  *Fn* subsp. *nucleatum* CW7c5  *Fn* subsp. *nucleatum* CW8 | CW7 containing pcarR  CW7 containing pcarR-3F  CW7 containing p3F  CW7 containing pCWU6-P*fruR(P1)-luc*  CW7 containing pCWU6-P*fruR(P2)-luc*  ∆*carS*; an isogenic derivative of CW1 | | | This study  This study  This study  This study  This study  (2) |
| *Fn* subsp. *nucleatum* BG20 | ∆*fruR*; an isogenic derivative of 23726 | | | This study |
| *Fn* subsp. *nucleatum* BG21 | *hp(2-31),* a hypothetical gene upstream of *fruR*  an isogenic derivative of 23726 | | | This study |
| *Fn* subsp. *nucleatum* BG21c1 | BG21c1 containing p*Hp* | | | This study |
| *Fn* subsp. *nucleatum* BG21c2 | BG21c1 containing p*Hp_his6_* | | | This study |
| *Fn* subsp. *nucleatum* BG22 | CW8 containing pZP07-*radD* | | | This study |
| *A. oris* MG1 | Wild Type strain | | |  |
| *S.oralis #34* | Wild Type strain | | |  |
| *S.gordonii* DL1 | Wild Type strain | | |  |
|  |  | | |  |
| *E. coli* DH5a  *E. coli* BL21(DE3) | Cloning host  Protein expression host | | |  |
| **Plasmids** |  | | |  |
| pCWU6 | *E. coli*/*Fusobacterium* shuttle vector, chloramphenicol /thiamphenicol resistance; cm^R^/thia^R^ | | | (1) |
| pBCG02 | Deletion plasmid with HicA as the selection marker | | | (3) |
| pCarR | pCWU6 expressing CarR under its original promoter | | | This study |
| pCarR-3F | pCWU6 expressing CarR-3XFLAG under *carR*’s original promoter | | | This study |
| p3F | pCWU6 expressing CarR-3XFLAG under *carR*’s original promoter | | | This study |
| pMCSG53-carR | expressing CarR-3XFLAG in *E. coli* | | | This study |
| pBCG02-∆*hp* | suicide plasmid for deletion of *hp* (2-31) | | | This study |
| pBCG02-∆*fruR* | suicide plasmid for deletion of *fruR* | | | This study |
| pHp | pCWU6 expressing Hp under its putative original promoter | | | This study |
| pHp_His6_ | pCWU6 expressing Hp-His6 | | | This study |
| pCWU6-P*fruR(P1)-luc* | *luc* gene expression was controlled by *fruR(P1)* promoter | | | This study |
| pCWU6-P*fruR(P2)-luc* | *luc* gene expression was controlled by *fruR(P2)* promote | | | This study |
| pZP05 | pCM-galK contains oriFn-repA rregon from pCWU6 | | | (4) |
| pBCG11 | pZP05 without galK and its promoter | | | This study |
| pRadD_23726_ | pZP07 expression the whole length of *radD* _23726_ under P*fdx* promoter | | | This study |
| pRadD_21_A_ | pZP07 expression the whole length of *radD* _21_1A_ under P*fdx* promoter | | | This study |
|  | |  |  | |
|  | |  |  | |
|  | |  |  | |
|  | |  |  | |

**Table S2:** Primers and DNA oligos used in this study

| **Primer** | | **Sequence^1^** | | | | **Use for** | | |
| --- | --- | --- | --- | --- | --- | --- | --- | --- |
| pCG02-pcarR3xF-F | | | TTACGAATTCGAGCTCGCTGCAGAGGTACCGTGAAAAAATATGTTGTAATTTTAAGTGGAAA | | | pCarR & pCarR-3F | | |
| pCG02-pcarR-R | | | AAAAGCTTGAGATCTGCTCGAGCTATTATTATTCATCTTCTTTAAGAACATAGCCTAAAC | | | pCarR & pCarR-3F | | |
| pCG02-CarR-3XF-R1 | | | CTTATCATCATCATCCTTATAATCTATATCATGATCCTTATAATCTCCATCATGATCCTTATAATCTTCATCTTCTTTAAGAACATAGCCTAAAC | | | pCarR-3F | | |
| pCG02-CarR-3XF-R2 | | | AAAAAGCTTGAGATCTGCTCGAGCTATTACTTATCATCATCATCCTTATAATCTATATCA | | | pCarR-3F | | |
| pCG02 P(carR) R | | | CATGATCCTTATAATCTTCATCTTCCATTATATCACCTTCTTACATATATTTTAATT | | | p3F | | |
| pCG02- P(carR) F  RACE outer primer  RACE inner primer  *radB* outer R  *radB* inner R  S-pCM-galK-F | | | GAAGATGAAGATTATAAGGATCATGATGG  GCTGATGGCGATGAATGAACACTG  CGCGGATCCGAACACTGCGTTTGCTGGCTTTGATG  TCATAATATTCTCTTTTTTCTTCTA  TATATTATTTGCACTGTTAGTACATG  GCTAAATTCAATTACACCTAAATTAT | | | p3F | | |
| pMCSG53(5kb)-F | | | ATTGGATTGGAAGTACAGGTTCTCGG | | | pMCSG-CarR | | |
| pMCSG53(5kb)-R | | | ATTGGAAGTGGATAACGGATCCG | | | pMCSG-CarR | | |
| EX-carR-F | | | GAACCTGTACTTCCAATCCAATAAAATTTTAGTAGTTGAAGATG | | | pMCSG-CarR | | |
| EX-carR-R | | | GGATCCGTTATCCACTTCCAATctatTCATCTTCTTTAAGAACATAG | | | pMCSG-CarR | | |
| HpupF | | | CCTAGGCCATGGCATATGGACTGCTGGAAGTGTTTCGATC | | | pBCG02--∆*hp* | | |
| HpupR | | | TATCTCTTCCGCATTTTGGAGCTCTGGAA | | | pBCG02--∆*hp* | | |
| HpdnF | | | TCCAAAATGCGGAAGAGATATGTTATTTGAAGACAGAAT | | | pBCG02--∆*hp* | | |
| HpdnR | | | CTTGACGAGTTCTTCTGAGCCATGCTCTGCTTTATCTACAAGAT | | | pBCG02--∆*hp* | | |
| fruB-up-F(SalI) | | | ggcgagtcgacGTTAGCACCTCCTCAATATACTTTTTTTAC AAT | | | pCM-galK--∆*fruB* | | |
| fruB-up-R(kpnI) | | | ggcga GGTACC TGAAAATCCT TTACTCTAAC AATAAAGTCA ATG | | | pCM-galK--∆*fruB* | | |
| fruB-dnF(kpnI) | | | ggcga GGTACC GCTTGTGGAA CAGCAACAAG TTTCTCAGAG GAT | | | pCM-galK--∆*fruB* | | |
| fruB-dnR(SacI) | | | ggcg t gagctc TAGTAAACTG AATATTGGAT AGATTACCAT AG | | | pCM-galK--∆*fruB* | | |
| PfruR-R3 | | | GGATTCAAAGTTACTGAATATATCATATCTCTTCCTTTTGTATATATTGATTATAATA | | | *pfruB* | | |
| Com-fruB-F | | | TCAATATATACAAAAGGAAGAGATATGATATATTCAGTAACTTTGAATCCCTCCATT | | | *pfruB* | | |
| Com-fruB-R | | | GTATAAATGTTAGTGATGATTATAAAAAATCTTTAATTTCCATAATTCTCCTTTTCTATAA | | | *pfruB* | | |
| Pfdx-F | | | CCTAGTTTATTTTTTTAGCACCATCAGCTAGTGTAGTAGCCTGTGAAATAAGT | | | pRadD | | |
| Pfdx-R | | | TTATTTTATTCCTGCCCGGAACCTATAGTGAGTCG | | | pRadD | | |
| Com-radD-F | | | CACTATAGGTTCCGGGCAGGAATAAAATAAGAGGGG | | | pRadD | | |
| Com-radD-R | | | TCGCTTTTAATTCTTCTAAAGTCTTTTTAAGTTCTAGAAGTCTTACCTGGAG | | | pRadD | | |
| pBCG11-F | | | TTAAAAAGACTTTAGAAGAATTAAAAGCG | | | pRadD | | |
| pBCG11-R | | | TAGCTGATGGTGCTAAAAAAATAAACTAGG | | | pRadD | | |
| pfruR-F | | | GAAACAGCTATGACATGATTACGAATTCGAGCTGTTAGCACCTCCTCAATATACTTTTTTTAC | | | pCWU6-P1-luc | | |
| pfruR-R | | | ATTTTCTTCACGTTCTGTTTCCATATCTCTTCCTTTTGTATATATTGATTAT | | | pCWU6-P1-luc | | |
| Luc-F | | | ATAATCAATATATACAAAAGGAAGAGATATGGAAACAGAACGTGAAGAAAAT | | | pCWU6-P1-luc | | |
| Luc-R | | | GTATAAATGTTAGTGATGATTATAAAAGTATAAATGTTAGTGATGATTATAAAATGCTTGG | | | pCWU6-P1-luc | | |
| pfruR-R2 | | | ATTTTCTTCACGTTCTGTTTCCATATCTCTTCCTTTTGTATATATTGATTATAGTAGATGATGATCTTCAATAAGGCTACTGCGACATCCATTAATGTTGAGAG | | | pCWU6-P2-luc | | |
| pHp-R | | | GTATAAATGTTAGTGATGATTATAAAATCCATATCTCTTCCTTTTGTATATATTGATTATA | | | pHp | | |
| galKrem-F | | | caaccatatgTAAACATAGCTGATGGTGCTAAAAAAATAAACTAGG | | | pZP07 | | |
| Galkrem-R | | | catatggttgGTTAAAAAGACTTTAGAAGAATTAAAAGCG | | | pPZ07 | | |
| pZP07-F | | | TTAAAAAGACTTTAGAAGAATTAAAAGCG | | | pRadD | | |
| pZP07-R | | | TAGCTGATGGTGCTAAAAAAATAAACTAGG | | | pRadD | | |
| pradA-F | | | TATGACCATGATTACGAATTCGAGCTGGATTGACTTTTGTAAGAAACAGG | | | pP*radA*-*radD* | | |
| pradA-R | | | cacgttctgtttcCATAAATTCAACCTCCCCTTTTT | | | pP*radA*-*radD* | | |
| Luc-F2 | | | GTTGAATTTATGgaaacagaacgtgaagaaa | | | pP*radA*-*radD* | | |
|  | | |  | | |  | | |
| **RT-PCR** | | |  | | |  | | |
| RT-radD-F | | | GCAGCAGCACCAACAATAAAT | | |  | | |
| RT-radD-R | | | GGTGCTTCAGGAGGTGTTATC | | |  | | |
| RT-radB-F | | | GAAGAGGCTGAAACAGAAAAAG | | |  | | |
| RT-radB-R | | | CTTCTTGCCTTGCTTTTTTAGC | | |  | | |
| RT-radA-F | | | GAACTTTCTGAAAGAGCAGCA | | |  | | |
| RT-radA-R | | | CTTTCTTCTGGAGCTGCTAAC | | |  |  |  |
| RT-fruB-F | | | GGTGAAACTAACAGAGCCTATGAA | | |  | | |
| RT-fruB-R | | | CTGCTCTATAAATGTTCCTGTAAATC | | |  |  |  |
| RT-gyrB-F | | | CACCATTTGGTGTGGGAAATAG | | |  | | |
| RT-gyrB-R | | | TTCCTCTTCCATTGTCCATAACT | | |  |  |  |
| C4N14_00100 RT-F | | | GGAATCTTTGTTAAACGTCAAGG | | |  | | |
| C4N14_00100 RT-R | | | GTGAAAAAGTTCTTACCCCTTCT | | |  | | |
| C4N14_01790 RT-F | | | CACTTATGATAGTAGCAAGTAAAGC | | |  | | |
| C4N14_01790 RT-R | | | CACCTTTAAAAAGGCTTCCATTG | | |  | | |
| C4N14_03355 RT-F | | | GAACTTGCCAGAGAAGGTAAG | | |  | | |
| C4N14_03355 RT-R | | | GTTTTACCAACTCCAGGTTCA | | |  | | |
| C4N14_05075 RT-F | | | GTTTTCAATGAAGAAGGGGTTAC | | |  | | |
| C4N14_05075 RT-R | | | GCTTTTGCCAAAGTTGAAAGT | | |  | | |
| C4N14_05365 RT-F | | | GATGGATCTTGATGATGATGGA | | |  | | |
| C4N14_05365 RT-R | | | GATGCTTTCATTTCATCTCCAT | | |  | | |
| WalR RT-F | | | GCAAGAATTAGAGTGGCAACA | | |  | | |
| WalR RT-R | | | GACAACCCCTTGTTTTTCATGA | | |  | | |
| Fap2-RT-F | | | GCTCATGGCAATTTGGAATGA | | |  | | |
| Fap2-RT-R | | | GTTTTCAGCCATATCCATAGCA | | |  | | |
| **EMSA primers** | | | |  | |  | | |
| radABCD EMSA F-Cy5 | | | | CGAGAGACAAAAGCATTGTAATATAG | |  | | |
| radABCD EMSA F | | | | CGAGAGACAAAAGCATTGTAATATAG | |  | | |
| radABCD EMSA R | | | | CTCATCATTTGCAAATATTCCAATAG | |  | | |
| FruRBA EMSA F-Cy5 | | | | CCATCCATTAATGTTTCCAGAGC | |  | | |
| FruRBA EMSA F | | | | CCATCCATTAATGTTTCCAGAGC | |  |  |  |
| FruRBA EMSA R | | | | CTGTCTTCAAATAACATATCTCTTCC | |  | | |
| megL EMSA F-Cy5 | | | | CACAGTGTACTATGACAGTTTTTAG | |  | | |
| megL EMSA F | | | | CACAGTGTACTATGACAGTTTTTAG | |  | | |
| megL EMSA R | | | | CCTAAACCAGATTTTTTCATTTCC | |  | | |
| C4N14_09785(GLPV) EMSA F-Cy5 | | | | GAAATAAAATGAATAAAAAAACATTAATAATTTATATAATTAATAATAA | |  | | |
| C4N14_09785(GLPV) EMSA F | | | | GAAATAAAATGAATAAAAAAACATTAATAATTTATATAATTAATAATAA | |  | | |
| C4N14_09785(GLPV) EMSA R | | | | GTTTCTTTACTCATTCTAAATTCCTCC | |  | | |
| Kal (LDP) EMSA F-Cy5 | | | | | CAGTTTTGATGTTAGAAAACTGTAC |  | | |
| Kal (LDP) EMSA F | | | | | CAGTTTTGATGTTAGAAAACTGTAC |  | | |
| Kal (LDP) EMSA R | | | | | CATACCCTTACCTCTTATTTTCATTATATC |  | | |
| Kal (LDP2) EMSA F-Cy5 | | | | | CTTTAATTCATATTGAATAAGACTGGGC |  | | |
| Kal (LDP2) EMSA R | | | | | CTTTAATTCATATTGAATAAGACTGGGC |  | | |
| Fap2 EMSA F-Cy5 | | | | | CCTCTTGCAACTTTATGTATGG |  | | |
| Fap2 EMSA F | | | | | CCTCTTGCAACTTTATGTATGG |  | | |
| Fap2 EMSA F | | | | | GATTATTTCCcatTATAATTTCCCCC |  | | |
| **Competitive EMSA synthetized probe DNA** | | | | |  | |  | |
| pradA BS P1 | TCAGATGAATATTTTTGATGACATAGTATAAAAAAAATGATATAATGAAAACAGTTATATAAATTATATAT**ATATAC**AATAAATAAT**A** | | | | | | |  |
| pradA BS P2  pradA BS P3 | TCAGATGAATATTTTTGATGACATAGTATAAAAAAAATGATATAATGAAAACAGTTATA**ATATAC**AATAAATAAT**A**  TCAGATGAATATTTTTGATGACATAGTATAAAAAAAATGATATAATGAAAACAGTTATATCTGAGCACCAT**ATATAC**AATAAATAAT**A** | | | | | | |  |
| pradA BS P4 | TCAGATGAATATTTTTGATGACATAGTATAAAAAAAATGATATAATGAAAACAGTTATATGAATGCTATAT**ATAT**ACAATAAATAAT**A** | | | | | | |  |
| **DNAase footprinting primers** | | | | |  | | |  |
| pradA Footprinting FAM F | | | | | TATAAGGATTGACTTTTGTAAGAAACAGG | | |  |
| pradA Footprinting R | | | | | CTCATCATTTGCAAATATTCCAATAG | | |  |

*

**Table S3:** Nomenclature Variations of Genes in the rad Operon Over the Years

| **Year** | **Gene Nomenclature** | **Description** | **Reference** |
| --- | --- | --- | --- |
| **2009** | *radA-radB-radC-radD* (arginine-inhibitable adhesin) | The outer membrane protein RadD is identified as an arginine-inhibitable adhesin essential for interspecies adherence and biofilm architecture. | Kaplan et al., 2009. *Molecular Microbiology*, 71(1), pp.35-47. |
| **2016** | *radC* also called *fad-I* (*Fusobacterium-associated defensin inducer*) | Fad-I, a *Fusobacterium nucleatum* cell wall-associated diacylated lipoprotein, induces human beta defensin 2 through TLR-1/2 and TLR-2/6 signaling. | Bhattacharyya et al., 2016. *Infection and Immunity*, 84(5), pp.1446-1456. |
| **2020** | *radA*, *radB* renamed to *rapA, rapB* (*RadD-associated proteins*) | Redesignation of *radA* and *radB* as *rapA* and *rapB*, indicating their association with RadD in fusobacterial interspecies interaction and biofilm formation. | Shokeen et al., 2020. *Microorganisms*, 8(1), p.70. |

1. Wu C, Al Mamun AAM, Luong TT, Hu B, Gu J, Lee JH, D’Amore M, Das A, Ton-That H. 2018. Forward genetic dissection of biofilm development by *Fusobacterium nucleatum*: novel functions of cell division proteins FtsX and EnvC. MBio 9:10.1128/mbio. 00360-18.

2. Wu C, Chen Y-W, Scheible M, Chang C, Wittchen M, Lee JH, Luong TT, Tiner BL, Tauch A, Das A. 2021. Genetic and molecular determinants of polymicrobial interactions in *Fusobacterium nucleatum*. Proceedings of the National Academy of Sciences 118:e2006482118.

3. Gc B, Zhou P, Wu C. 2023. HicA Toxin-Based Counterselection Marker for Allelic Exchange Mutations in *Fusobacterium nucleatum*. Applied and Environmental Microbiology:e00091-23.

4. zhou p, GC B, Wu C. 2024. Development of a Conditional Plasmid for Gene Deletion in Non-Model *Fusobacterium nucleatum* strains. bioRxiv:2024.09. 09.612158.
